# An update on the effect of intra-articular intervention strategies using nanomaterials in osteoarthritis: Possible clinical application

**DOI:** 10.3389/fbioe.2023.1128856

**Published:** 2023-02-16

**Authors:** Soumayeh Amirsaadat, Halimeh Amirazad, Ramin Hashemihesar, Nosratollah Zarghami

**Affiliations:** ^1^ Stem Cell Research Center, Tabriz University of Medical Sciences, Tabriz, Iran; ^2^ Department of Medical Biotechnology, Faculty of Advanced Medical Science, Tabriz University of Medical Sciences, Tabriz, Iran; ^3^ Department of Histology and Embryology, Faculty of Medicine, Altinbas University, Istanbul, Türkiye; ^4^ Department of Medical Biochemistry, Faculty of Medicine, Istanbul Aydin University, Istanbul, Türkiye; ^5^ Department of Clinical Biochemistry and Laboratory Medicine, Faculty of Medicine, Tabriz University of Medical Sciences, Tabriz, Iran

**Keywords:** osteoarthritis, intra-articular, drug delivery, nanomaterials, pipeline

## Abstract

Osteoarthritis (OA) is the most common progressive condition affecting joints. It mainly affects the knees and hips as predominant weight-bearing joints. Knee osteoarthritis (KOA) accounts for a large proportion of osteoarthritis and presents numerous symptoms that impair quality of life, such as stiffness, pain, dysfunction, and even deformity. For more than two decades, intra-articular (IA) treatment options for managing knee osteoarthritis have included analgesics, hyaluronic acid (HA), corticosteroids, and some unproven alternative therapies. Before effective disease-modifying treatments for knee osteoarthritis, treatments are primarily symptomatic, mainly including intra-articular corticosteroids and hyaluronic acid, so these agents represent the most frequently used class of drugs for managing knee osteoarthritis. But research suggests other factors, such as the placebo effect, have an essential role in the effectiveness of these drugs. Several novel intra-articular therapies are currently in the clinical trial processes, such as biological therapies, gene and cell therapies. Besides, it has been shown that the development of novel drug nanocarriers and delivery systems could improve the effectiveness of therapeutic agents in osteoarthritis. This review discusses the various treatment methods and delivery systems for knee osteoarthritis and the new agents that have been introduced or are in development.

## 1 Introduction

Osteoarthritis (OA) is the most common joint disease. It can affect any joint, but mainly affects the hips, knees, hands, and feet in the body. OA is an inflammatory joint disease recognized by pathological features in bone, cartilage, muscle, synovium, periarticular fat, and ligaments, causing stiffness, joint dysfunction, loss of valued activities, functional limitations, and pain. Osteoarthritis is a very complex pathophysiological process and a multifactorial disorder, thus creating limited treatment options for OA. Age, sex hormone level, obesity, major joint injury, and genetics are important risk factors for OA. Patients with OA commonly suffer from comorbidities, and are more disabled. Lack of or constrained physical activity is responsible for higher age-related mortality rates (Katz et al., 2021). Osteoarthritic chondrocytes are deranged and degenerated, as evidenced by an uncoordinated gene expression pattern and ultrastructural changes. In addition, in OA progression, the whole joint is involved OA, commonly known as wear and tear disease, is the consequence of complex interactions between many elements such as genetic, metabolic, biomechanical, and biochemical factors (Liu-Bryan, 2013). To date, geneticists have identified 124 single nucleotide polymorphisms (SNPs) correlated with OA. These SNPs comprise 95 independent loci spread throughout the genome, with some loci (such as in the collagen type XI Alpha 1) containing multiple SNPs showing separate associations at the locus. Generally, OA is a common polygenic disease that occurs because of the inheritance of several risk alleles of moderate individual impact. Certainly, multiple disease-modifying OA drugs (DMOADs) are in clinical trials at present; for instance, Wnt inhibitors and intra-articular FGF-18 and TGF-β growth factor therapies, target proteins whose genes have been identified by genome-wide association studies (GWAS). Intra-articular (IA) drug delivery provides direct access to the joint and can relieve inflammatory symptoms. The prospect of Intra-articular (IA) drug delivery is exciting and has a number of advantages over systemic administration. As a disadvantage, the quick clearance of drugs from the joint is considered a severe obstacle to their therapeutic efficacy (Jones et al., 2019). Many researchers are developing drug delivery systems (DDS) or formulations with a slower release effect to increase drug retention capacity and reduce side effects. The present review article attempted to accumulate novel information in intra-articular drug delivery, gene and cell therapies, nanotechnology-based application in OA therapy especially drug delivery systems (DDS) for effective OA treatment, and finally the IA therapy pipeline. In this part of the review, we explain intra-articular drug delivery and cell therapies before describing DDS. We optimism that the ideas created in this review will support the improvement of effective OA treatment in the future.

## 2 Intra-articular drug delivery

It offers many pluses since Intra-articular (IA) drug delivery provides direct access to the joint, hence strengthening the local bioavailability of therapeutic drugs while decreasing potential adverse events, systemic exposure, and total expenses. However, IA injections are usually recognized as safe; the rapid clearance of drugs limits their therapeutic effect (March et al., 2014). In addition, factors such as systemic effects (Habib, 2009), administration technique (Jackson et al., 2002), and drug residence time contribute to treatment variability (Gerwin et al., 2006). Hyaluronic acid (HA) and corticosteroids are the most general agents administered by IA injection for joint lubrication and the management of pain (Ma et al., 2022). The effectiveness of IA therapies, such as HA and corticosteroids, is limited by their fast clearance. Therefore, we need safe formulations which provide extended and sustained drug availability. For this purpose, many synthetic and natural (bio) materials have been utilized to accomplish desirable qualities such as enhanced articular retention time with the steady and slow release of drugs, and drug delivery vehicles’ biodegradation (Rai and Pham, 2018).

### 2.1 The placebo effects

As a clinical event, the placebo effect explains how a sham medical intervention could improve a patient’s condition due to factors related to the patient’s perception of the medical intervention. There are many examples of placebo interventions, such as saline injections, sugar pills, and therapeutic rituals. The placebo effects are not only limited to inert interventions. Standard effective treatments can also create a placebo effect. Generally, the placebo effect is considered a confounding variable to control. However, there has been much interest in studying this phenomenon due to some significant research showing its potential to modulate treatment results in recent years (Munnangi et al., 2018). A systematic review investigated the placebo effect in knee osteoarthritis (KOA), which confirmed that the measured placebo effect was remarkably better than no treatment. Adapting significant clinical effects of therapies such as HA IA in real-world settings and normal clinical practice with measurable placebo effects on symptom reduction in clinical trials needs a rigorous method to better realize the placebo effect and other associated factors. For instance, since interventions of KOA may be performed using several approaches, such as IA injection, oral or topical, studies have been conducted to quantify alternative placebos’ effects, which showed that IA placebo shows more significant pain reduction compared with oral placebo. It is worth recalling that novel intra-articular injections may not be suitable for every patient (Fazeli et al., 2022).

### 2.2 Standard IA treatments

Corticosteroids and hyaluronic acid (HA) are the most common agents used through IA therapy. Albeit not definitively described as the standard of care, these two agents are used as standard treatment options for managing pain in KOA patients who are unresponsive to analgesics, non-pharmacologic therapy, and non-steroidal anti-inflammatory drugs (NSAIDs). IA injection of the knee might be a good option for patients who cannot tolerate oral medications when medicines are no longer effective and for patients avoiding or delaying surgery (Rastogi et al., 2016).

#### 2.2.1 Corticosteroids as pain relievers

IA corticosteroid injections of the knee are useful for short-to medium-term therapy of joint pain. Corticosteroids have both immunosuppressive and anti-inflammatory effects. Corticosteroids directly affect steroid hormone receptors and disrupt the immune response and the inflammation process at some levels (Bodick et al., 2015; Frederick et al., 2021). In that way, corticosteroids diminish the permeability of blood vessels and can prevent inflammatory cells accumulation, neutrophil superoxide production, metalloprotease, and their activators, as well as inhibit the production of multiple inflammatory mediators like leukotrienes and prostaglandin. The clinical anti-inflammatory effects of these actions reduce erythema, heat, swelling ng, and joints’ tenderness, and increase relative viscosity and HA concentration (Ayhan et al., 2014). There are many corticosteroids, such as dexamethasone LA, triamcinolone acetonide, methylprednisolone acetate, and betamethasone. The most frequently consumed corticosteroids include triamcinolone acetonide (TA) and methylprednisolone acetate (MA). Their usual dosage is 40 mg (Law et al., 2015).

#### 2.2.2 Hyaluronic acid

Hyaluronic acid (HA), officially identified as hyaluronate or hyaluronan, is an unsulfated glycosaminoglycan with high-molecular weight formed from the repetitive accumulation of molecular chains of N-acetyl-glucosamine and glucuronic acid. It is responsible for shock absorbency and joint lubrication during movements, enhancing synovial fluid viscosity. HA, which functions as a backbone for the proteoglycans of the extracellular matrix (ECM), creates a hydrated pathway by which the cells can move and migrate (Brockmeier and Shaffer, 2006). Studies have indicated that HA elevates chondrocyte proliferation and differentiation, which has aroused attention to its application in tissue-engineering techniques (Yagishita et al., 2005). HA contributes to the inhibition of enzymatic cartilage degradation (Bowden et al., 2017). In the arthritic joint, molecular weight and concentration of HA are reduced by 33%–50%, which restricts the effectiveness of HA to maintain joint biomechanics at normal levels. Visco-supplementation could provoke HA endogenous production and replace the lost HA (Strauss et al., 2009). Also, some clinical studies have displayed that IA-HA hampers pain and recovers joint function in OA patients. HA treatment has positive effects and is well tolerated (Park et al., 2021; In and Ha, 2022). Several studies focusing on the knee joint have revealed that repetitive treatment of the patients by IA-HA therapy, as a safe method, can delay total joint replacement surgery by up to 3 years (De Lucia et al., 2020).

### 2.3 IA delivery of bioagents targeting inflammatory mechanisms

**FIGURE 1:**
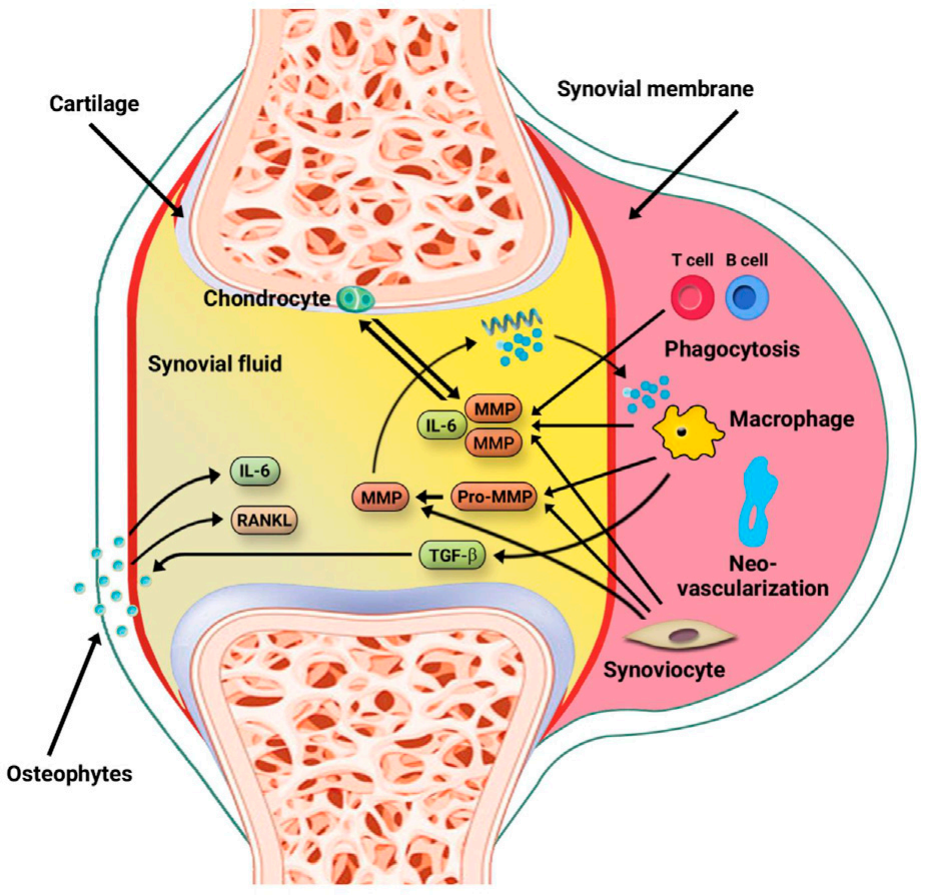
The secretion of cytokines in OA disease. This joint disorder consist of the secretion of cytokines by the cartilage, bone, and synovial membrane. IL-1β, IL-6, and TNF cytokines are produced by macrophages, T cells, chondrocytes, and osteophytes in response to damaged tissue. The released Pro-MMPs by macrophages and synoviocytes, undergo cleavage and turn into functional MMPs contributing to tissue injury. TNF, IL-6, and IL-1β enter the bloodstream, making OA as a systemic disorder. B and T cells in the synovial fluid are engaged by the cytokine milieu and contribute to local synovitis. Furthermore, some cytokines, primarily RANKL and IL-6, are released by bone cells (Chevalier et al., 2013).

Recently some evidence has shown that the progression of OA is related to an imbalance of anabolic and catabolic factors (Figure 1). These findings have created hope that biological agents may be utilized in OA therapy. Some clinical studies using IA biological agents have been discussed in the following subsections (Chevalier et al., 2013).

#### 2.3.1 Targeting TNF

As a potent pro-inflammatory cytokine, together with other cytokines, TNF acts as a catabolic factor for cartilage (Aletaha et al., 2011). It cooperates with chondrocytes, showing a relationship with knee cartilage loss (Stannus et al., 2010). TNF provokes MMPs release by synovial fibroblasts leading to cartilage destruction and diminishing chondrogenesis by the NF-kB pathway *via* downregulating the SOX9 production. Another particular function of TNF is to induce the apoptosis signal in chondrocytes. Also, TNF-alpha has been also shown to hinder mesenchymal stem cell differentiation into chondroblasts, which in turn affects chondrogenesis (Chisari et al., 2020). The joint inflammation caused by TNF-alpha activity has been investigated in patients with OA and rheumatoid arthritis (RA), illustrating it as a promising target to be considered in RA treatment. Currently, anti-TNF-alpha-targeted therapy is used as a treatment option for OA patients, which has shown satisfactory results in reducing inflammation. Several agents that target TNF-alpha, namely trastuzumab, etanercept, infliximab, and adalimumab, have been developed to treat OA. However, the mechanisms by which TNF-alpha regulates the inflammation in synovial fibroblasts from patients with OA, need to be further clarified (Li et al., 2018).

#### 2.3.2 Targeting IL-1β

IL-1β pro-inflammatory cytokine is also involved in the pathogenesis of OA. This cytokine, which seems to be related to cartilage destruction, is synthesized by mononuclear cells, chondrocytes, synovial tissues, and osteoblasts, induces the production of some catabolic and inflammatory factors. In OA patients, the level of IL-1β is upregulated in the synovial membrane, synovial fluid, subchondral bone, and cartilage. IL-1β could act in cooperation with other cytokines or independently to instigate and propagate inflammation (Thomas et al., 1991). Some studies have investigated the significance of targeting IL-1 signaling utilizing IL-1 Receptor Antagonist (Anakinra) or targeted gene therapy. The obtained results showed the significant protective effect of treatment in surgical models of rapidly progressive OA (RPOA) early after surgery. A similar result was observed in rats treated by anakinra following anterior cruciate ligament transection (ACLT) (Vincent, 2019).

### 2.4 Growth factor therapy: Targeting cartilage metabolism

Growth factors are small peptide molecules that can provoke cell growth, differentiation and, division processes. Multiple growth factors in articular cartilage, act together to control homeostasis and articular cartilage development during life. So, growth factors are also considered new therapeutic targets for enhanced cartilage repair in articular cartilage defects or conditions with extensive cartilage loss like OA (Fortier et al., 2011). Various growth factors are involved in bone repair, but two essential families can be categorized as follows: bone-derived growth factors (BMPs family) and autologous blood-derived growth factors which are generally applied for the regeneration of bone, and another family (Civinini et al., 2013).

### 2.5 Cell therapies

Lack of study comparability, internal limitations, and methodological make it difficult to critically evaluate the efficacy of cell therapies. However, given their widespread clinical use, a basic understanding of cell therapy is essential. In the sections below, an overview will be provided concerning the most common cell therapies used and/or studied for the treatment of KOA.

#### 2.5.1 Platelet-rich plasma

Platelet-rich plasma (PRP) is described as a portion of the liquid section of autologous blood with a higher concentration of platelet than the baseline. RP treatments have been applied for different indications for more than three decades, resulting in significant attraction in the PRP potential in regenerative medicine (Everts et al., 2020). Several companies have presented PRP preparation systems that allow outpatient and intraoperative use of PRP for orthopedic pathologies (Hall et al., 2009). PRP comprises a diverse and complex milieu of chemical mediators interacting with endogenous cells in the joint. Although IA injections of PRP can be recommended for patients with OA, however, this is not an FDA-approved approach (Dhillon et al., 2012; Beitzel et al., 2015).

#### 2.5.2 Bone marrow aspirate concentrate (BMAC)

Mesenchymal stem cells (MSCs), as multipotent cells, have the ability to differentiate into multiple cell types depending on environmental factors, so they can participate in bone and soft tissue healing (Amirazad et al., 2022). The main site for the storage of MSCs is bone marrow. The use of MSCs in soft tissue and bone healing has displayed favorable results; one of the few intraoperative concentrated stem cell transfer methods approved by the FDA (Glenn et al., 2021). Bone marrow aspirate concentrate (BMAC) is a cell therapy approach used in KOA therapy. The benefit of this method is its composition of various cell types, such as MSCs, monocytes, and platelets (Kouroupis et al., 2020).

BMAC is prepared from bone marrow aspirate, usually aspirated from the ilium crest, by means of density gradient centrifugation (DGC). BMAC is demonstrated to contain high levels of MSCs, platelets, hematopoietic stem cells (HSCs), cytokines, and chemokines, including TGF-β and PDGF (Themistocleous et al., 2018). Clinically, MSCs and BMAC hold a therapeutic promise in several orthopedic conditions, such as KOA and spinal OA. Nevertheless, the quality of clinical implications remains poor (El-Kadiry et al., 2022).

#### 2.5.3 Stromal vascular fraction (SVF)

**FIGURE 2:**
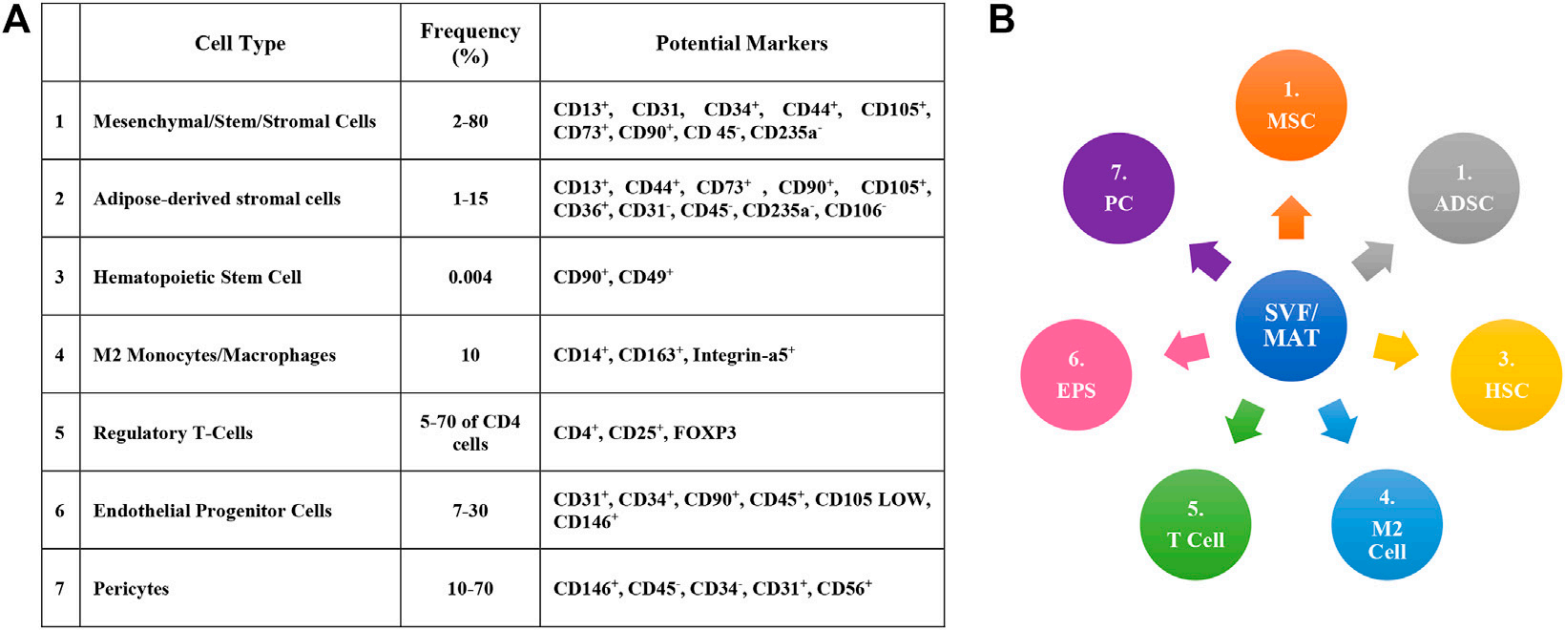
**(A)** Different cells that form SVF and MAT-SVF (micronized adipose tissue-stromal vascular fractions). **(B)** Cells, associated markers and frequency of occurrence of SVF and MAT-SVF (Ude et al., 2021).

SVF is a collection of variable cell populations obtained through liposuction derived by enzymatic digestion of lipoaspirate. It includes endothelial progenitors, hematopoietic cells, endothelial cells, ASCs, pericytes, adipose progenitors, macrophages, immune cells, fibroblasts, leukocyte subtypes, smooth muscle cells, lymphatic cells, and other uncharacterized cells (Figure 2) (Ude et al., 2021). Studies have shown that about 2% of isolated SVF cells expressed hematopoietic associated CD45^+^ and CD34^+^ and 7% expressed mesenchymal CD146+ and CD105+. The expression pattern of markers for SVF-derived cells is similar to bone marrow-derived mesenchymal stromal cells (BM-MSCs), such as CD105/SH2, CD29, CD90, CD71, SH3, and CD44, along with downregulation of CD45, CD31, and CD24 (Han et al., 2015). The ASCs in SVF are between <1 and >15%; nevertheless, they could be significantly varied in alignment with the patient’s health, age, and the method used for harvesting (Shimozono et al., 2019). Unlike the treatment by ADSCs, which requires *in vitro* expansion, there is no need for such a thing through treatment by SVF (Ashammakhi et al., 2019). Other benefits of SVF are heterogeneous cell composition, which contribute to better outcomes (Pak et al., 2018). Moreover, the presence of pericytes in the SVF, by differentiating to active MSCs in response to inflammation and injury, plays a critical role in regeneration (Michalek et al., 2017). In recent years, some studies have showed the short and mid-term results of SVF for KOA, displaying their joint function improvement and analgesic effect (Hong et al., 2019; Tran et al., 2019; Garza et al., 2020). Zhang et al. (2022) conducted a medium-term study on SVF treatment in KOA patients. The results showed that up to 5 years after SVF treatment, almost 60% of patients had an acceptable clinical status (Aubourg et al., 2021; Xiao et al., 2022).

### 2.6 Adipose tissue injections

In recent years, the attention to using adipose tissue to treat orthopedic disorders has been raised. Adipose-derived stromal/stem cells (ASCs), a group of MSCs, are obtained from adipose tissue located in the perivascular blood vessels and capillaries within adipose tissue. Studies have shown that compared to BMAC, ASCs are outnumbered per unit volume, less sensitive to senescence secondary caused by culture expansion, and more rapidly proliferate in culture (Malanga and Ibrahim, 2017; Dadashpour et al., 2018). Previously, ASCs were separated utilizing digestive enzymes from the suspensions of the stromal vascular fraction. This method was challenging, not only because of the complex harvesting techniques but also because of regulatory concerns about cell manipulation and expansion. Therefore, the FDA updated the guidelines for using all stem cell therapies on 16 November 2017 (Malanga and Bemanian, 2019). Some research has illustrated the benefits of ASCs in improving knee joint function and pain. Bistolfi and colleagues examined the safety and efficacy of autologous concentrated adipose tissue as a treatment method for patients with KOA. In this study, the knees of 20 OA patients were IA injected with autologous ASCs. Patients’ articular pain and functionality were evaluated by VAS (visual analog scale) and WOMAC (Western Ontario and McMaster Universities Osteoarthritis Index) scores at 3, 6, and 18 months from the infusion. The result of treatment was safe, and all patients reported improved pain relief and increased function (Roato et al., 2019).

#### 2.6.1 Mechanisms involved in the therapeutic properties of MSCs

Since the initial discovery of MSCs in 1960, they have been the subject of scientific research. MSCs are described as cells that have the capacity to differentiate into multiple lineages of mesoderm, such as osteoblasts, chondrocytes, adipocytes, and hematopoietic stroma. These cell types have been considered in cell therapy due to their immunomodulatory potential, a tendency to damage/inflam tissues, and relatively easy isolation and expansion. There are many sources for MSCs in the body, including adipose tissue, bone marrow, placenta, umbilical cord, cord blood, dental pulp, amniotic fluid, endometrium, lung tissue, skeletal muscle tissue, dermal tissue, liver tissue, and many of them have been utilized in clinical studies (Spees et al., 2016).

**FIGURE 3:**
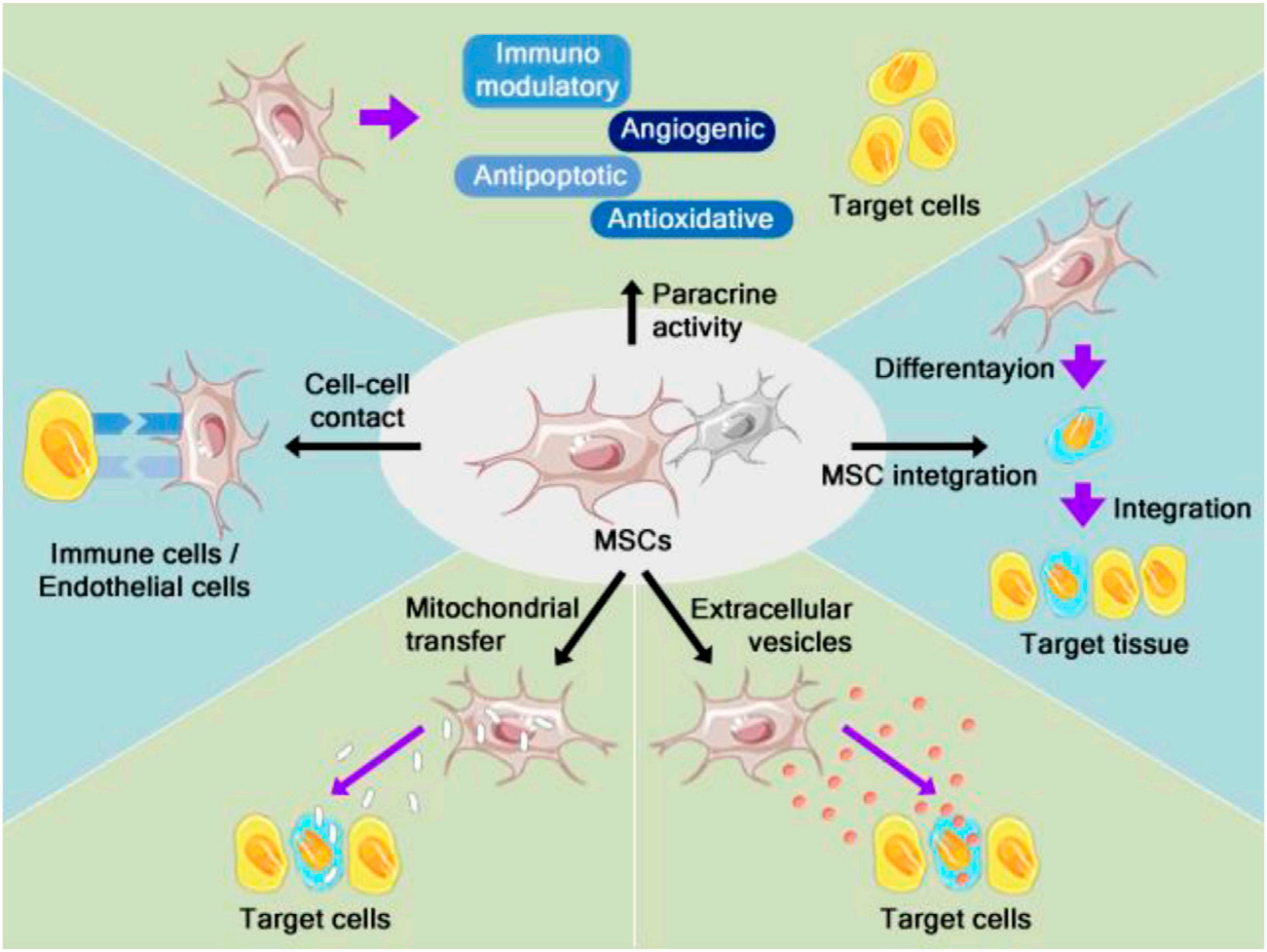
The mechanisms of action in therapeutic procedures based on MSCs. MSCs heal and/or rescue injured cells *via* differentiation into replacement cells and modifying immune responses. The ability of MSCs to function as immunomodulators is exerted by cell-cell contact and interaction with immune cells (Fan et al., 2020).

Two main aspects show the capacity of MSCs as therapeutic options: replacement of damaged tissue and immunomodulatory function. The pleiotropic effect is the main mechanism underlying MSC therapy and allots the release of different soluble factors that exhibit immunomodulatory, antioxidant, angiogenic, and anti-apoptotic effects (Figure 3). The immunomodulatory and regenerative effects of MSCs are modulated through cell-cell interactions mediated by tunneling nanotubes to targeted cells. Furthermore, an anti-inflammatory effect was observed by releasing exosomes containing several microRNAs (miRNAs) that increase the cellular proliferation during the regeneration of bone tissue (Merimi et al., 2021; Sahabi et al., 2022).

### 2.7 Gene therapy and gene delivery system

Gene therapy, by providing therapies for underlying factors rather than disease symptoms, illustrates the great potential in modern medicine. Gene delivery is an important part of gene therapy. However, delivering a therapeutic gene to target cells *via* crossing the plasma membrane remains a major limitation in gene delivery. Hence, for safe and highly efficient delivery of nucleic acid to the target site, it is a prerequisite to study the vector/carrier that is necessary to transport the nucleic acid across the negatively charged and hydrophobic cell membrane. An optimized carrier/vector that effectively compresses and provides stability until the nucleic acid is transported to the target site in the cells and transfers the nucleic acid in the nucleus *via* crossing the cell membrane should be considered extra-cellular barriers (such as mechanisms of DNA degradation), and intracellular barriers (such as nuclear and intracellular uptake, endosomal escape, DNA release) present in the cell system. Gene-based systems primarily use adenoviruses, lentiviruses, and retroviruses, that are unable to replicate (change to replication-deficient), and they are only able to nucleic acid delivery and expression. Constant expression of therapeutic genes is the main advantage of these systems, but they have some limitations, such as toxicity, lack of optimization, and immunogenicity. Non-viral gene delivery systems are classified as chemical methods (using natural or synthetic carriers) and physical methods (such as ultrasound, microinjection, and hydrodynamic applications). Liposomes, polymers, inorganic materials, and dendrimers are applied for the non-viral gene delivery system. This system has some advantages, such as easy modification, cell/tissue targeting, and low immune response. However, the main challenge is increasing gene transfection efficiency into cells. Exosomes are important intracellular messengers, so they can be utilized as delivery vehicles for transferring drug and genetic material. Synovial mesenchymal stem cell-derived exosomes can induce chondrocyte proliferation. As sleep is beneficial for cartilage restoration, and also, circular RNAs (circRNAs) have been indicated to be involved in the OA pathogenesis, Sleep-Related circRNA (SR-circRNA) cartilage repair was the first time screened employing melatonin therapy and small extracellular vesicles (sEVs) transferring SR-circRNA (circRNA3503) were constructed. A triblock copolymer gel was utilized as a carrier for sEVs. *In vitro* studies have illustrated that this system has the ability to promote chondrocyte regeneration and decrease the progressive loss of articular cartilage, and it is an effective therapy for preventing OA progression. Lipid-based nanocarriers can deliver RNA or DNA into cells. Sometimes these particles are trapped *via* the endocytosis process, and the release of nucleic acids is limited. To solve this challenge, another nanocarrier (HA-coated p5RHH) called cytolytic peptide was proposed, which was improved to decrease its pore-forming capability and maintain its capability to cross a bilayer membrane. The improved peptide can form a self-assembled nanostructure, and then stabilization *via* HA, the siRNA can be rapidly transported to the cytoplasm and suppress the expression of specific genes *in vitro* and *in vivo*. Delivery of this nanocomposite to human cartilage explants suppresses *β*-Catenin/WNT3a signaling, leading to decreased chondrocyte apoptosis. Moreover, the relationship between genetics and epigenetics of OA can open up the option as a therapeutic approach. A number of OA risk loci colocalize with genes that encoding histone deacetylase (HDAC) inhibitors and histone modifying proteins indicate effect as inhibitors of catabolic molecules expression, such as IL-1 and matrix metalloproteinases (MMPs), in the mouse OA models and OA chondrocyte. Also, CRISPR-Cas9, as a promising tool, can modify DNA methylation (DNAm). In the functional study, a dCas9-TET1 construct was utilized for the demethylation of the hypermethylated mQTL in the RWDD promoter. The resulting increase in RWDD2B expression reversed the effect of OA genetic risk in this locus. Although this study was performed in an immortalized cell line, it highlights the possibility of applying epigenome editing to counteract the gene expression impacts of a risk source. The initiation of CRISPR-Cas9 and the following progress of the Cas9 toolbox has revolutionized targeted editing of the genome and epigenome. Besides CRISPR dCas9-DNMT3a/TET1 as a novel tool was utilized in OA genetic studies (Aubourg et al., 2021; Grigsby et al., 2021; Xiao et al., 2022).

## 3 Nanotechnology-based application in OA therapy

As an indispensable tool in medicine and research, Nanotechnology by using various fields of study, including biology, chemistry, physics and electronics, plays an important role in the development of new approaches by focus on the manipulation of particles (molecules, atoms and macromolecules) with size of 1–100 nm (Nejati-Koshki et al., 2017; Jeevanandam et al., 2018; Hu et al., 2020). These types of particles, which are called nanoparticles, because of their particularity in the scale structure, possess some distinctive features, such as size and quantum effects as well as interfacial phenomena. The diversity of these properties related to the size makes nanoparticles favorable in new functions, and their manipulation and control may lead to the appearance of novel biological, physical and chemical characteristics. Besides, due to perfect scale for catalysis, high ratio of surface to volume, and availability of nanoscale structures in the body, nanotechnology has become a more important tool in science (Jin, 2020). To fabricate nanoparticles by nanotechnology, two common strategies are used, including top-down and bottom-up approaches. The first approach includes the nanofabrication tools that help to produce nanoparticles by the reduction of macro-sized structures. The latter approach involves chemical and physical processes that are used to integrate atomic or molecular constituents into larger particles in nanoscales (Baig et al., 2021).

Nanotechnology is widely used in various industries and medicine, even in clinical applications, such as Doxil and Ferumoxytol, which are effective in the treatment of ovarian cancer and iron deficiency anemia, respectively (Fathi Karkan et al., 2017; Farjadian et al., 2019). However, despite the incredible advancements in the usage of nanotechnology, it has not found its way into clinical application for OA treatment. By the way, recent studies have evidenced the significance of nanotechnology in the treatment of OA by developing drug delivery approaches. These systems have been shown to improve the specific targeting and increase the efficiency of drug delivery and therapeutic effects, reduce the side effects, extend the drug retention and circulation time, and inhibit the dispersion and degradation of drugs in body fluids (Corciulo et al., 2020; Guo et al., 2022). Subsequently, recently various delivery systems based on nanotechnology have been developed for OA therapy.

### 3.1 Improving joint drug delivery by nanotechnology

**FIGURE 4:**
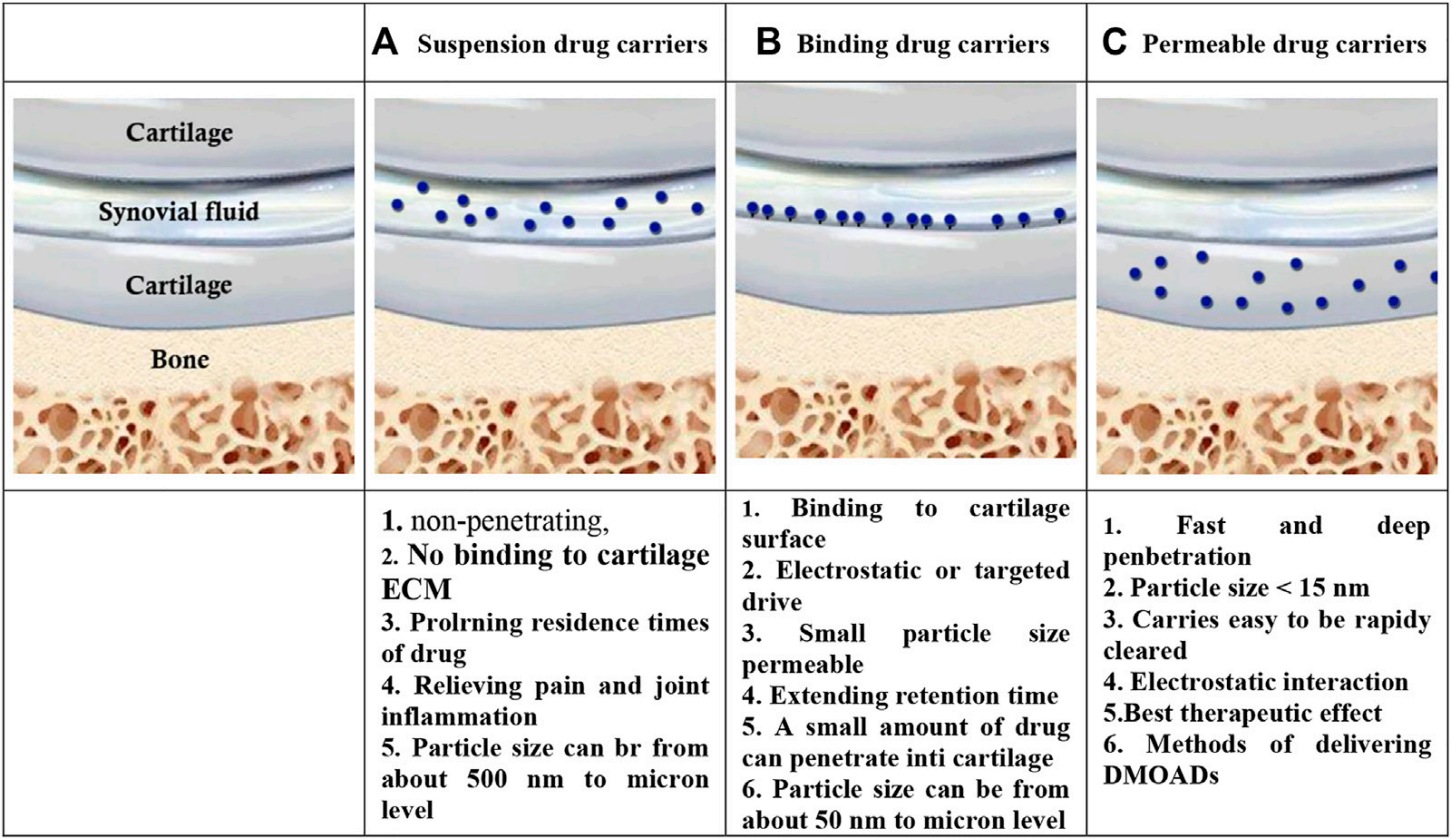
Three types of micro and nano drug carriers [**(A)**: Suspension drug carriers, **(B)**: Binding drug carriers, and **(C)**: Permeable drug carriers] and their characteristics (Huang et al., 2022).

Direct IA injections of therapeutic agents are generally employed to overwhelm the low rate of joint bioavailability seen in systemic administration but the rapid clearance of drugs limits their therapeutic effect and has been detected in various substances from small-molecule drugs to macromolecules, and among animal species (Larsen et al., 2008; Evans et al., 2014). Many researchers are developing drug delivery systems (DDS) or formulations with a slower release effect to increase drug retention capacity and reduce side effects. The frequently used DDS for IA injection is micro/nano DDS due to its good safety, easy modification feature, and sustained release performance. Suspension, binding, and permeation are three micro/nano-drug carrier types (Figure 4) (Huang et al., 2022). Suspension and binding carriers could resolve the obstacles related to short half-life and rapid clearance of therapeutic agents in the joint cavity. In addition, they significantly enhance the curative effect of therapeutic agents. Although, ECM is only lost at International Cartilage Repair Society (ICRS) grade III-IV when the suspension and binding carriers can well demonstrate the efficacy of drugs that promote MSCs differentiation and proliferation or affect chondrocytes. In OA patients suffering from grade I-II ICRS, ECM is rather undamaged, so a tiny amount of the drugs released from carriers could penetrate the cartilage; also, the depth of penetration is not satisfactory. Hence, the drug at the target site cannot reach the therapeutic concentration, thereby compromising therapeutic efficacy (Zeng et al., 2021; Huang et al., 2022). In the following, some types of drug carriers were introduced.

### 3.2 Liposomes

Liposomes are known as one of the favorable drug delivery systems and the first FDA-approved nano-drug carrier. Liposomes are spherical vesicles with an aqueous core surrounded by a phospholipid bilayer with an approximate size between 50 and 5,000 nm. Three types of liposomes are commonly formulated, including unilamellar vesicles, large unilamellar vesicles, and multilamellar vesicles. These structures are very maneuverable and they can be modified through coating other polymers, such as antibodies to have become immunoliposomes (Nsairat et al., 2022). Until now, some valuable liposome-based delivery systems have been formulated for medical ends, for example: Liprostin and Doxil which are used for the treatment of thrombosis and cancers (Gu et al., 2020; Pala et al., 2020). More importantly, Lipotalon^®^ (dexamethasone palmitate) is another liposome-based drug delivery system being used for IA therapy of OA (Evans et al., 2014). Besides, some studies showed that liposomes could be effectively used in the delivery of therapeutic agents in OA. As mentioned, it has been shown that the encapsulation of adenosine and its receptor A2A, as factors involved in cartilage homeostasis, in liposomal carriers and their delivery using the IA method in obese mice and rats suffering from OA, could consider the progression of the disease (Corciulo et al., 2020). Besides, liposomes have been also shown to be very useful in the formulation of drug delivery carriers for Rapamycin. This drug, which is an inhibitor of mTOR, a potential therapeutic target of OA, through encapsulation in the liposomes and IA delivery can effectively reduce the inflammation in the spontaneous OA guinea (Chen et al., 2020). Fish oil protein (FP) is another therapeutic agent that is used for OA treatment because of its anti-inflammatory effect. Sarkar et al. (2019) evidenced that gold nanoparticles (GNP) tagged with fish oil could be encapsulated in dipalmitoyl phosphatidylcholine (DPPC) liposomes (FP-GNP-DPPC) and delivered to OA rat models using the IA strategy. They observed that these drug carries by the constant release of FP-GNP into the synovial fluid, leding to the downregulation of Bax, Caspase 3, and p53 apoptosis genes and pro-inflammatory cytokines as well as increased the expression of antioxidant, such as superoxide dismutase (SOD) and glutathione reductase (GSH). These results suggested the great potential of FP-GNP-DPPC for treatment of OA. The infiltration M1 macrophages into the joint synovium in one of mechanisms involved in obesity-induced OA. It has been shown that the encapsulation for clodronate into liposomes and its IA delivery in obese mice models, could deplete M1 macrophages and reduce collagen X, leading to the suppression of OA progression (Feng et al., 2011; Ponzoni et al., 2018). In addition, Curcumin, as a herbal compound involved in the inhibition of human cancers by its anti-inflammatory effects (Farajzadeh et al., 2018; Tavakoli et al., 2018; Mansouri et al., 2020), has been shown through encapsulation into soybean phosphatidylcholines liposomes and IA delivery could exerts efficient suppressive effects on OA progression in interleukin-1 *β* induced *in vitro* models. This was because of the increased bioavailability of Curcumin through encapsulation into liposomal carriers (Yeh et al., 2015). Overall, these findings suggest that liposomes could serve as useful carriers for better and specific IA delivery of therapeutic agents of into the region of action and lead to more favorable outcomes through treatments.

### 3.3 Exosomes

Exosomes are nano-sized (50–150 nm in diameter) phospholipid bilayer vesicles that are derived from the plasma membrane. These structures, which are biologically released from normal and pathologic cells, carry various cargoes, including proteins, DNA, and RNA molecules (Doyle and Wang, 2019). Exosomes derived from some specific cells, such as MSCs, have been shown to exert therapeutic effects in various diseases, including OA (Cai et al., 2020). In particular, exosomes derived from BMSCs and chondrocytes have been evidenced to carry non-coding RNAs, including miRNA and lncRNA, that prevent the expression of inflammatory factors and proteolytic enzymes, subsequently inhibiting OA progression (Miao et al., 2021). As an example, MSC-derived exosomes were reported to contain miRNAs that are involved in the regulation of genes participating in signalling pathways activated in OA, such as the Wnt/*β*-Catenin pathway, SIRT1/p53 pathway and NF-kB pathway (Jin, 2020). Exosomes could also be fused with other compounds for a more specific delivery. To be mentioned, Liang et al. (2020) fused exosomes derived from chondrocytes with lysosome-associated membrane glycoprotein 2b containing chondrocyte-affinity peptide (CAP) to overcome the challenge in drug delivery through the dense cartilage matrix. They showed that the delivery of the fused exosomes containing CAP and miR-140 could remarkably decrease OA development in rat models. Despite the mentioned advantage of exosomes to be used in OA treatment, their low production rate by MSCs and other cells is considered a main challenge in clinical applications. By the way, currently, a large number of studies are being carried out to increase the yield of exosomes, which could open new avenues into the exosome-based treatment of OA (Nguyen et al., 2021).

### 3.4 Chitosan

As a polyaminosaccharide, chitosan is obtained from the N-deacetylation of the natural polysaccharide chitin. Due to its biodegradability, non-toxicity, biocompatibility, and mucoadhesive and bacteriostatic properties, chitosan received attention in numerous pharmaceuticals, biomedical, drug slow-release material, food, and environmental fields. Chitosan nanoparticles (NPs) have been extensively studied as gene delivery systems and nanocarriers for drugs and proteins (Muxika et al., 2017; Saeed et al., 2020; Serati-Nouri et al., 2020). Kang and colleagues developed thermo-responsive polymeric nanospheres, which in response to temperature change offer an independent and simultaneous dual drug delivery capacity. Nanospheres based on chitosan conjugated Pluronic F127 (PF127) grafting carboxyl group were designed for simultaneous delivery of diclofenac (DCF) and kartogenin (KGN) to treat OA. The nanospheres demonstrated sustained release of KGN and immediate DCF release, which were separately regulated *via* the change in temperature. They also stimulated chondrogenic differentiation of MSCs, which was improved by cold shock treatment. Results showed that the nanospheres have chondroprotective and anti-inflammatory effects and could suppress the OA progression in rat models (Kang et al., 2016).

### 3.5 Polyester amide (PEA)

**FIGURE 5:**
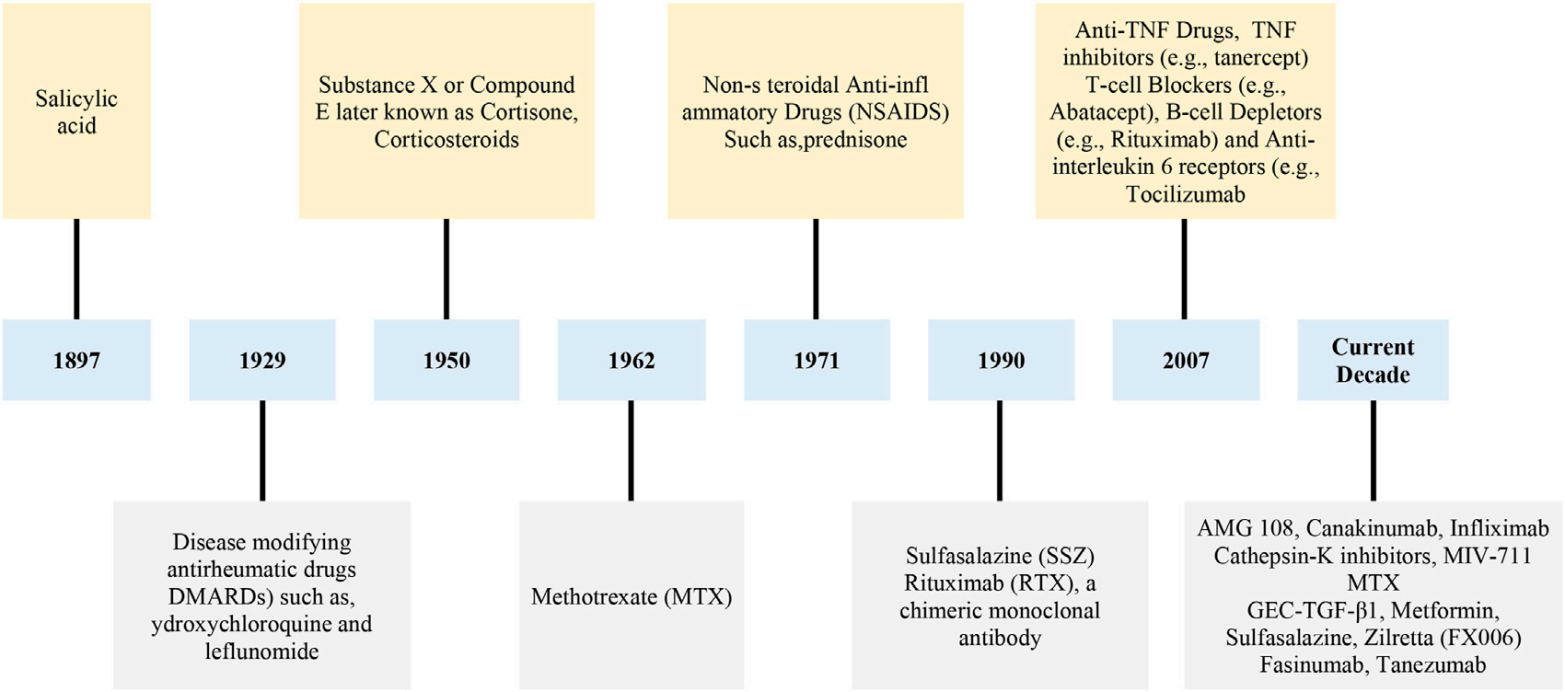
Timeline of treatments in the history of osteoarthritis (Hyndman, 2017; Kraus et al., 2018; Pelechas and Karagianni, 2020; Cai et al., 2021).

The PEAs are synthetic biodegradable polymers containing amide and ester groups in their chain. They can be produced by polycondensation of linear monomers or ring-opening polymerization of cyclic monomers. This chemical structure improves the materials’ biodegradability, mechanical properties, and processability. In addition, the most important property of PEAs is their degradable composition through the surface erosion mechanism (Andrés-Guerrero et al., 2015; Winnacker and Rieger, 2016). Janssen et al. (2016) studied the capacity of celecoxib-loaded PEA microspheres as a self-regulating DDS for treating KOA-related pain. Their results showed after a primary rapid release of celecoxib load on day one (about 15%), the drug was then gradually released over 80 days. Histologically, IA biocompatibility of PEA-microspheres was demonstrated, whereas there were no cartilage damage and necrosis or synovial thickening after injections. The degradation of PEA-microspheres was considerably higher in OA-induced knee joints in comparison with the contralateral healthy knee, however loading of celecoxib on PEA microspheres remarkably prevented the degradation, demonstrating a DDS with self-regulatory action. The mentioned study evidenced that celecoxib-loaded PEA microspheres have a great capacity to utilize as a safe DDS with suitable biocompatibility, long-term retention in the joint cavity, and self-regulatory property for OA treatment. The timeline of treatments in the history of osteoarthritis is summarized in Figure 5.

### 3.6 Poly-lactic-co-glycolic acid (PLGA)

PLGA is one of the polymers used in DDS construction and formulation for therapeutic approaches due to its remarkable characteristics, including biosafety, biodegradability, biocompatibility, and versatility in functionalization (Ghitman et al., 2020). PLGA polymers have an extensive degradation range which is tuned *via* their copolymer and the ratio of their molecular weight. PLGA can dissolve in common solvents, such as chlorinated, acetone, and ethyl acetate solvents (Operti et al., 2021). Supplements, PLGA-based nanocarriers ensure optimal drug bioavailability by protecting it from premature degradation, providing sustained and target-specific delivery, accelerating intracellular penetration of the drug, and lessening the side effects (Ghitman et al., 2020). Goto et al. (2017) developed fluvastatin-loaded PLGA microspheres (FLU-PLGA) to achieve controlled IA administration of fluvastatin to treat OA. First, *in vitro* experiments were conducted, and the results showed that the drug could continuously be released within 4 weeks. After that, the therapeutic effects of FLU-PLGA were investigated in a rabbit OA model. The knees were subdivided into four groups. Five weeks after IA injection, it was histologically demonstrated that the OARSI scores were lower in the FLU-PLGA-treated group. The study showed that a single IA injection of FLU-PLGA could be a promising new therapeutic method for the management of OA patients. Some properties of these carriers are summarized in Table 1.

**TABLE 1:** Comparison of different nanovechicles from various aspects.

Carrier	Mmune response	Stability and other propeties	Circulation	Ref.
Liposome	Inhibiting monocyte production and depletion M1 Macrophage	Natural-based liposomes (phosphatidylcholine species: bilayer structures) are permeable and have low stable properties.	The intermediate sized liposomes (150–200 nm) have the longest circulating time.	Nakhaei et al. (2021)
		Saturated-phospholipids-based liposomes (dipalmitoyl phosphatidylcholine) are rigid and impermeable (Sterols, such as ergosterol, stigmasterol, and cholesterol).		
Exosome	Exhibit pro-inflammatory activity at the initial stage of transplantation to activate the innate immune system and subsequently exhibit anti-inflammatory activity.	Stable, Natural generation, Low immunogenicity, Have capability to cross biological barrier	Indeterminate loading efficacy, rapid clearance from blood, and weak targeting capability.	Chen et al. (2021b), Xian Bo et al. (2022)
Chitosan	Has excellent anti-inflammatory and antioxidants capability the IL-6, IL-10, and TNF-α plasma levels were down-regulated	Low toxicity, low immunogen, high biodegradable, high biocompatible, stable	Rapid clearance from systemic circulation, but PEGylated chitosan nanoparticles have long circulation time in the blood.	Mohyuddin et al. (2021)
PEA	Inhibits the level of inflammatory cytokines	High biodegradable, high biocompatible	Slow clearance from systemic circulation	Kropp et al. (2014)
		Non-toxic, high stable		
		Good thermal and mechanical properties		
		Extended degradation and release profiles in comparison with PLGA		
PLGA	Modulating monocyte recruitment.	High biodegradable, high biocompatible, non-toxic	Tunable prolonge blood circulation time	Rezvantalab et al. (2018), Chatterjee and Chanda (2022)
		Stable (have a high residence time in the joint cavity)		
		The drug release from PLGA can be controlled by regulation the ratio of glycolic acid (GA) and lactic acid (LA) in the PLGA chain.		
		(50 GA: 50 LA in PLGA): 2 months		

## 4 The IA therapy pipeline

Currently, many IA-based therapeutic strategies for OA are in clinical development. Some of these treatments are discussed in the following sections. Clinical Trials of IA-based therapeutic for the treatment of osteoarthritis are outlined briefly in Supplementary Table S1.

### 4.1 LMWF-5A (Ampion)

Human serum albumin has been for treating shock and burns for more than three decades, considering its advantageous tolerability and safety. Ampion is a fraction of commercial human serum albumin with molecular-weight lower than 5,000 Da containing aspartyl-alanyl diketopiperazine that may function as an immunomodulator and exert anti-inflammatory effects (McGrath, 2015). *In vitro* studies showed the involvement of Ampion in the modulation of the inflammatory immune response by regulating a pathway involving T cells (Shimonkevitz et al., 2008).

The Ampion effect on pain reduction in KOA was studied, and its primary results were published. This observation focuses on the prominent aspects of this trial in a heterogeneous “real-world” group of KOA patients. In this study, patients were divided to receive a single 4- or 10-mL IA injection of Ampion or saline as the control group. At baseline, the age of patients was an average of about 62 years (64% female and 36% male). WOMAC pain scores of Ampion-treated patients were remarkably better than those of placebo-treated patients at week 12. The Ampion effect on pain was even more noticeable in patients suffering from acute KOA: the assessed treatment difference from the control group was −0.42. The adverse event profile was generally slight and similar in patients receiving Ampion (41%) and saline (47%). This clinical trial showed that Ampion was safe and effective in relieving moderate to severe KOA pain 12 weeks after administration by IA injection into the knee (Bar-Or et al., 2014).

### 4.2 HA-triamcinolone hexacetonide (Cingal)

Cingal is a new product developed to provide short-term pain relief from a corticosteroid, triamcinolone hexacetonide, with the persistent pain relief of a HA viscosupplement. It is a single IA injection with the total volume of 4 mL consisting of 18 mg of triamcinolone hexacetonide and 88 mg HA. This trial aimed to indicate the safety and efficacy of Cingal for relieving joint pain and symptoms in KOA patients. A double-blind, multicenter saline-controlled trial compared the utilization of saline, HA, and Cingal in 69, 150, and 149 patients with KOA, respectively. The WOMAC score at 26 weeks suggested that Cingal provided better symptom relief in this trial than the placebo. Nevertheless, Cingal only showed statistically remarkable advantages compared to HA alone in the first and third weeks (Hangody et al., 2018).

### 4.3 JTA-004

JTA-004 is a novel protein solution, which originated from plasma and supplemented with clonidine and HA, developed by Bone Therapeutics S.A., Belgium. The intra-articular administration of JTA-004 has been shown to ease the local discomfort and pain related to IA injections *via* the short-term analgesic effects of clonidine in OA patients, and, and to restores joint homeostasis thanks to the interaction between human plasma and HA. After injection into the knee joint, the jellification is induced by plasma through a coagulation cascade, forming a coagulable gel that results in a three-dimensional network stabilized through interactions between the patient’s synovial proteins and HA fibers (Gentili et al., 1996; Sun et al., 2014; Martin-Alarcon and Schmidt, 2016). This gel exhibits a rheological and mechanical behavior similar to synovial fluid with shock-absorbing and lubricating effects and protects the patient’s cartilage. To examine the safety and efficacy and to choose the most satisfactory formulation, a single IA administration of three JTA-004 formulations, which differ in clonidine concentration and volume, was tested in comparison with Hylan G-F 20 reference treatment for 6 months. Based on the interim results, the JTA-004 formulation containing 20 mg hyaluronic acid and 200 μg clonidine was selected at 6 months. The difference in WOMAC score from baseline at month six was 9.49  mm; therefore, the superiority of JTA-200 was not statistically indicated. There were no significant changes in adjusted mean difference from baseline between JTA-200 and the control group in terms of physical function, pain, total WOMAC score, stiffness subscales, and wellbeing score at any time point. However, JTA-200 stimulated greater enhancements in WOMAC scores compared to Hylan G-F 20 treatment. Besides, the safety of IA injection of JTA-004 was evidenced in KOA patients through this study (Bettonville et al., 2021).

### 4.4 PTP-001

PTP-001 is a lyophilized, sterile, human placental tissue preparation in the development phase as a novel agent for the therapy of OA symptoms and structural pathology. Unlike other conventional autologous therapies (e.g., ASCs, BMAC and PRP), PTP-001 is a room-temperature (“off-the-shelf”) stable therapeutic agent being resuspended in saline instantly before use. In a study, Flannery et al. (2021) characterized the biochemical features of PTP-001 in OA progression using interesting periclinal *in vitro* and *in vivo* models. PTP-001 contains multiple beneficial substances, such as growth factors, anti-inflammatory molecules, and cytokines. Then, PTP-001 was demonstrated to dose-dependently inhibit the production of MMP-13 by chondrocytes, as well as diminish proinflammatory cytokine secretion from macrophages/monocytes. PTP-001 also enhanced synovial cell proliferation and remarkably decreased pain responses over 6 weeks post-dosing, in rat OA models. The duration and magnitude of pain relief after an IA injection with PTP-001 were comparable to rats treated with corticosteroid (active control). A significant reduction in cartilage histopathology scores was obtained for animals treated twice with PTP-001. These results demonstrated that PTP-001 is a promising biologic therapy for OA that may participate in disease modification and symptom management by a multimodal mechanism.

### 4.5 Adalimumab

Adalimumab (Humira^®^) is a recombinant, human IgG1 monoclonal antibody that specifically blocks TNF-α and prevents the binding of TNF-α to p75 and p55 receptors and neutralizes cytokine activity (Bang and Keating, 2004). Adalimumab can affect biological responses that are regulated *via* TNF-α. For instance, adalimumab is correlated with changes in the concentration of molecules responsible for leukocyte emigration (e.g., ELAM-1, ICAM-1, and VCAM-1) (Plosker and Lyseng-Williamson, 2007). A randomized, open-label trial investigated the efficacy and safety of adalimumab compared with HA *via* IA injection for moderate to severe KOA. 56 patients with moderate to severe KOA were randomly divided into HA or adalimumab groups. On day 0, in the adalimumab group, patients received adalimumab (10 mg), while the other group received HA (25 mg). All patients received 200 mg/day of celecoxib for 4 weeks. At baseline, the pain VAS, Physician Global Assessment (PhGA), Patient Global Assessment (PGA), and WOMAC scores were similar between groups. The reduction in WOMAC score, VAS score, and WOMAC physical function score from baseline to the fourth week was more significant in the adalimumab than in the HA group. A greater reduction in the PhGA and PGA scores from baseline to week four was noticed in the adalimumab than the HA group. There was no difference in terms of side effects between two groups. These results illustrated that adalimumab by IA injection was tolerated and effective for moderate to severe KOA (Wang, 2018). However, other studies failed to observe clinically significant improvements in patients who received short-term adalimumab treatment (Aitken et al., 2018; Maksymowych et al., 2022).

### 4.6 rhFGF18 (Sprifermin)

Fibroblast growth factors (FGFs) are associated with a wide array of biological processes, such as cell growth, morphogenesis, differentiation, inflammation, angiogenesis, tumor growth, tissue repair and multiple developmental processes. The FGF family consists of 23 members that signal by four FGF receptors. Some of the FGFs have been investigated for their therapeutic capacity. Recombinant human FGF18 (rhFGF18) is evaluated as a treatment for OA (Sieber and Gigout, 2020). A randomized, double-blind, placebo-controlled study of rhFGF18 in patients with advanced KOA was conducted to estimate the safety of IA rhFGF18 and to assess systemic exposure, histology, biomarkers, and other cartilage parameters. Patients were randomly divided 3:1 to rhFGF18 or placebo, injected into the knee once or once a week for 3 weeks, and followed up for 6 months. 55 patients were treated with rhFGF18, 25 with a single dose, 30 with multiple doses, and 18 received a placebo. There was no significant difference between the placebo and active groups in incidence, severity, and side effects. No significant difference was seen between placebo and rhFGF18 in physician-assessed local tolerability, swelling, or pain in the knee. No meaningful differences between treatment groups, or changes over time, were seen for ECG or safety laboratory parameters. This trial showed no serious safety concerns; however, more extensive studies are needed. The positive effects of rhFGF18 on histological and other parameters in KOA also warrant further investigation (Dahlberg et al., 2016).

### 4.7 Fasinumab (REGN475)

As a human recombinant IgG4 monoclonal antibody, Fasinumab binds specifically to NGF without affecting signaling pathways mediated by other neurotrophins, including brain-derived neurotrophic factor and neurotrophin-3 (NT-3). A double-blind, randomized, placebo-controlled exploratory trial in KOA patients was conducted to evaluate the safety, efficacy, and tolerability of fasinumab. In this study, 217 patients (40–75 years old) were randomized 1:1:1:1 to receive intravenous fasinumab and a placebo on the first and 57th days. Daily pain intensity was recorded utilizing a numerical rating scale. Tolerability and safety were assessed as primary study endpoints through treatment-emergent adverse events (TEAEs). The endpoints of the secondary study included the change from baseline in walking knee pain and assessing function, pain, and stiffness employing the WOMAC index. After 6 months, the most common TEAEs included hyperesthesia, arthralgia, myalgia, joint swelling, and peripheral edema. TEAEs leading to discontinuation occurred in 3.7% of placebo patients and 5.6% of fasinumab patients. Results illustrated that all doses of fasinumab were correlated with significant improvements (*p* < 0.05) in WOMAC total and walking knee pain and subscale scores compared with placebo. Generally, fasinumab was well tolerated and correlated to an improvement in function and a remarkable reduction in walking knee pain for up to 8 weeks (Tiseo et al., 2014).

### 4.8 EP-104IAR

EP-104IAR is a novel IA formulation of corticosteroid fluticasone propionate (FP), which is developed to ease pain in OA patients and consists of FP crystals that are covered with polymer polyvinyl alcohol (PVA). A randomized, double-blind, placebo-controlled trial was carried out to measure the safety, efficacy, and pharmacokinetics (PK) of EP-104IAR in KOA patients. 32 patients were randomized (11 men, 21 women, mean age: 64.8 years), received a single dose of EP-104IAR or placebo, and were followed up for 42 weeks. The results showed that the well toleration of EP-104IAR by patients. In addition, average serum cortisol levels did not illustrate any clinically relevant deviation in comparison with the placebo group and stayed at the normal range of cortisol levels. Compared to marketed FP products, plasma pharmacokinetics (PK) concentrations were in good safety margins. The assessment of efficacy displayed that EP-104IAR offered fast improvement in OA symptoms, and the effects were consistently sustained for 2–3 months across all measures (Malone et al., 2021; Hunter et al., 2022).

### 4.9 TPX-100

TPX-100 is a peptide derived from the matrix extracellular phosphoglycoprotein (MEPE). MEPE is highly expressed *via* osteocytes cells, is downregulated in OA, and may have a role in OA bone remodeling. A study was conducted to evaluate the efficacy of TPX-100-5 in patients with bilateral KOA. 104 patients (25–75 years old) were divided to receive TPX-100 or a placebo (Leiman et al., 2020). The placebo-treated contralateral knee of each patient served as a paired control. Compared with placebo-treated knees, TPX-100-treated knees indicated a statistically significant reduction in pathologic bone shape change at 6 and 12 months. The correlation between total and medial tibiofemoral cartilage thickness changes and bone shape change was statistically significant in the TPX-100 group at 12 months (Leiman et al., 2020; McGuire et al., 2021).

## 5 Conclusion and prospective

KOA is the most common degenerative joint disease and represents a considerable social burden. IA injections of HA, corticosteroid, and PRP represent treatment options with minor side effects for pain and symptom relief in patients who do not respond to non-pharmacological treatments, analgesics, or NSAIDs and can delay surgical treatment. Currently, many new drugs are being developed that have shown good therapeutic potential and promise new approaches to treat KOA. However, the benefits of new agents should be carefully weighed against their potential risks and cost. Further studies are needed to assess new molecules and associated therapies in KOA IA injection treatment. This review summarized the significance of nanomaterials in the development of the delivery systems and improving the therapeutic effect of these agents for osteoarthritis through targeted delivery, biocompatibility and controlled release of drugs. However, in this case, there is also a need for further investigation to optimize these methods for clinical practice. Additionally, osteochondral lesions are usually great defects that require a significant amount of nanomaterials, and mass production of the nanomaterials is difficult, which is a challenge in osteochondral restoration. Currently, gene therapy technology is very popular, and numerous therapeutic targets for OA disease have been suggested, providing a theoretical basis for utilizing NPs in gene therapy. So there are logical reasons to be optimistic that data from genetic studies of OA and from genomic analyzes, which complement these, will be used for patient therapy. Based on the possible developmental origin of several OA risks, translation of genetic discoveries requires consideration of the time in a person’s life when it is best to initiate treatment. In the future, the application of enhanced chondrogenic potential cells combined with 3D models of cartilage, and cartilage with bone, will prepare more realistic and robust organ and cell models for functional analysis (FA) of OA SNPs and target genes. Moreover, the combination of the new nanotechnology with RNAs in OA defects may help to increase the success ratio of optimum therapeutic results (Jin, 2020). Nano-technology-based treatments such as smart nanobots as drug delivery systems, artificial intelligence, and three-dimensional printing methods can be used in the regeneration of osteochondral defects in the future (Deng et al., 2019).

## Glossary

ACLT: anterior cruciate ligament transection
ASCs: adipose-derived stromal/stem cells
BMAC: bone marrow aspirate concentrate
CAP: chondrocyte-affinity peptide
DCF: diclofenac
DGC: density gradient centrifugation
DPPC: dipalmitoyl phosphatidylcholine
ECM: extracellular matrix
FGFs: fibroblast growth factors
FP: fish oil protein
FP: fluticasone propionate
GNP: gold nanoparticles
GSH: GSHand glutathione reductase
HA: hyaluronic acid
HSCs: hematopoietic stem cells
IA: intra-articular
ICRS: international cartilage repair society
KGN: kartogenin
KOA: knee OA
MEPE: matrix extracellular phosphoglycoprotein
miRNAs: microRNAs
MSCs: mesenchymal stem cells
NPs: nanoparticles
NRS: numeric rating scale
NSAIDs: non-steroidal anti-inflammatory drugs
OA: osteoarthritis
PEA: polyester amide
PhGA: physician global assessment
PK: pharmacokinetics
PRP: platelet-rich plasma
PtGA: patient global assessment
PVA: polyvinyl alcohol
RA: rheumatoid arthritis
rhFGF18: recombinant human FGF18
SOD: superoxide dismutase
SVF: stromal vascular fraction
TEAEs: treatment-emergent adverse events
TEAEs: treatment-emergent adverse events
VAS: visual analog scale
