# Supplementary material for: An update on the effect of intra-articular intervention strategies using nanomaterials in osteoarthritis: Possible clinical application
Source: Front Bioeng Biotechnol. 2023 Feb 16;11:1128856. doi: 10.3389/fbioe.2023.1128856 (PMC9978162; doi:10.3389/fbioe.2023.1128856)
Supplement: Supplementary file 1 [file Table1.docx]

Supplementary Material

**An Update on the Effect of Intra-articular Intervention Strategies Using Nanomaterials in Osteoarthritis: Possible Clinical Application**

Soumayeh Amirsaadat^1^, Halimeh Amirazad^1,2^, Ramin Hashemi^3^ , Nosratollah Zarghami^4,5*^

*** Correspondence:** Corresponding Author: Zarghami[@tbzmed.ac.ir](mailto:davaran@tbzmed.ac.ir)

# Supplementary Tables

| TABLE 2.\| Clinical Trial of IA-based therapeutic for the treatment of osteoarthritis. | | | | | |
| --- | --- | --- | --- | --- | --- |
| Biological / chemical compound for therapy | **Condition** | **Phase** | **ClinicalTrials.gov Identifier** | **Recruitment Status** | **Ref.** |
| LMWF-5A (Ampion) | The Evaluation of the Efficacy / Safety Of An IA Of Ampion™ (AP-003-B) In Adults With Pain With KOA | III | NCT02556710 | C | [89] [87]  [110] [111] |
|  | An Efficacy and Safety Study of Two Doses of IA Ampion Injection for Pain of KOA | II | NCT01839331 | C |  |
|  | Study of Ampion for the Treatment of Pain and Function in Patients With Severe KOA. | III | NCT03988023 | C |  |
|  | AP-007 Study to Evaluate Safety and Exploratory Efficacy of Three IA Injections of Ampion in the Knee of Adults With Pain Due to OA | I/II | NCT02184156 | C |  |
|  | An Open Label Extension Assessment of the Safety of Long-Term Treatment With Ampion for Severe KOA | III | NCT03349645 | T |  |
|  | AP-008 Multiple Injection Study Evaluating Safety and Efficacy of Ampion in OA | III | NCT02242435 | C |  |
|  | AP-003-C Study to Confirm the Efficacy of Ampion™ in Adults With Pain Due to Severe KOA | III | NCT03182686 | C |  |
|  | AP-004-A Randomized Controlled Study to Assess Efficacy and Safety of IA Ampion for OA Pain in Knee | III | NCT02024529 | C |  |
| Cingal | Study of Cingal™ for the Relief of Knee OA Compared to Triamcinolone Hexacetonide at 39 Weeks Follow up (Cingal17-02) | III | NCT03390036 | C | [90]  [112] |
|  | Study of Cingal® and Triamcinolone Hexacetonide for the Relief of KOA Pain | III | NCT04231318 | A |  |
|  | Cingal Study for KOA | NA | NCT01891396 | C |  |
|  | Repeat Injection of Cingal® for KOA | III | NCT02381652 | C |  |
|  | Study of Cingal™ for the Relief of KOA Compared to Triamcinolone Hexacetonide | III | NCT03191903 | C |  |
|  | Early Effect of Cingal® Compared to Monovisc® in Patients With KOA (EEFFEK) | NA | NCT03062787 | C |  |
| JTA-004 | Phase III Study on the Safety and Efficacy of a Single IA Administration of JTA-004 in Symptomatic KOA (JTA-KOA2) | III | NCT04333160 | C | [94] [113] [114] |
|  | A Study on Visco-antalgic IA Administration in Symptomatic KOA | II/III | NCT02740231 | C |  |
| PTP-001 | Efficacy and Safety Trial of PTP-001 (MOTYS) for Symptomatic KOA | II | NCT05100225 | A | [99] [124] [125] |
|  | Safety Study of PTP-001 for Treating KOA | I | NCT04632966 | A |  |
| Adalimumab | KOA Inflammation and the Effect of Adalimumab (OKINADA) (OKINADA) | II | NCT02471118 | C | [102] [104] [103] [126] |
|  | Adalimumab for Inflammatory OA | I/II | NCT00686439 | C |  |
| TABLE 1 \| (Continued) Clinical Trial of IA-based therapeutic for the treatment of osteoarthritis. | | | | | |
| Biological / chemical compound for therapy | **Condition** | **Phase** | **ClinicalTrials.gov Identifier** | **Recruitment Status** | **Ref.** |
| rhFGF18 (sprifermin) | A Multicenter Study of rhFGF 18 in Patients With KOA Not Requiring Surgery | I | NCT01033994 | C | [106] [127] |
|  | Study of AS902330 (rhFGF-18) Administered IAly in Patients With Knee Primary OA Who Are Candidates for Total Knee Replacement | I | NCT00911469 | C |  |
|  | A Study to Investigate the Safety and Effectiveness of Different Doses of Sprifermin in Participants With KOA (FORWARD) | II | NCT01919164 | C |  |
| Fasinumab | A Study of the Safety and Efficacy of REGN475(SAR164877) in Patients With KOA | I/II | NCT00944892 | C | [107] [128]. |
|  | Long-Term Safety and Efficacy Study of Fasinumab in Patients With Pain Due to OA (OA) of the Knee or Hip (FACT LTS & OA) | III | NCT02683239 | C |  |
|  | A Study to Determine the Safety and the Efficacy of Fasinumab Compared to Placebo and Naproxen for Treatment of Adults With Pain From KOA or Hip (FACT OA1) | III | NCT03161093 | C |  |
|  | Study to Determine the Safety and the Efficacy of Fasinumab Compared to Placebo and Nonsteroidal Anti-inflammatory Drugs (NSAIDs) for Treatment of Adults With Pain From KOA or Hip (FACT OA2) | III | NCT03304379 | C |  |
|  | Study of REGN475 in Patients With Pain Due to KOA or Hip | II/III | NCT02447276 | C |  |
|  | Study to Assess Arthroplasty Specimens for KOA and Hip | II | NCT03949673 | T |  |
|  | A Study of the Safety and Efficacy of Subcutaneously Administered REGN475 in Patients With KOA | II | NCT01239017 | W |  |
|  | To Assess the Patients' Ability to Self-Administer Fasinumab (FACT DEVICE) | I | NCT03491904 | C |  |
|  | Study to Assess the Effects of Fasinumab on Peripheral Nerve Function in Patients With Pain Due to OA of the Hip or Knee | II | NCT03691974 | C |  |
|  | Assess the Efficacy and Safety of Fasinumab in Patients With Moderate-to-Severe Chronic Low Back Pain and OA of the Hip or Knee (FACT CLBP 1) | III | NCT03285646 | T |  |
| EP-104IAR | Safety Study of a Long-Acting Injectable Steroid to Treat KOA (STEPUP) | I | NCT02609126 | C | [108] |
|  | Study to Assess the Efficacy and Safety of EP-104IAR in Patients With KOA | II | NCT04120402 | R |  |
| TABLE 1 \| (Continued) Clinical Trial of IA-based therapeutic for the treatment of osteoarthritis. | | | | | |
| Biological / chemical compound for therapy | **Condition** | **Phase** | **ClinicalTrials.gov Identifier** | **Recruitment Status** | **Ref.** |
| TPX-100 | A Study Evaluating the Safety and Efficacy of a Second Course of TPX-100 in Subjects Who Previously Received TPX-100 for Patellar OA Involving Both Knees | II | NCT02837900 | C | [129] [109] |
|  | A Study Evaluating the Safety and Efficacy of IA Injections of TPX-100 in Subjects With Mild to Moderate Patello-Femoral OA Involving Both Knees | II | NCT01925261 | C |  |
|  | A Prospective Observational Study to Assess Long-term Changes in Cartilage Morphology in Subjects Who Previously Received TPX-100 or Placebo in Study TPX-100-1 for Patellar OA Involving Both Knees | - | NCT03125499 | C |  |
| Curcumin | Exploratory Non-Comparative Study to Assess the Efficacy of Highly Bioavailable Curcumin (Flexofytol) in Patients With KOA | I | NCT01909037 | C | [130] |
|  | The Efficacy and Safety of Curcuma Domestica Extracts and Ibuprofen in KOA | III | NCT00792818 | C |  |
| MM-II | Safety and Efficacy of an Injectable Medical Device to Treat KOA | I/II | NCT01365260 | C | [131] |
|  | Efficacy and Safety of MM-II for Treatment of Knee Pain in Subjects With Symptomatic KOA | II | NCT04506463 | A |  |
| Elixcyte | Adipose-derived Stem Cells (ADSCs) for KOA | I/II | NCT02784964 | C | [132] |
| GXCPC1 | Allogeneic Adipose Tissue-Derived Mesenchymal Stem Cells (GXCPC1) for KOA | I/II | NCT03943576 | R | [133] |
| Canakinumab | A Safety and Efficacy Study of Anti-inflammatory (Canakinumab) and Cartilage Stimulating (LNA043) Drugs Injected Into the Knee Joint of Participants With KOA | II | NCT04814368 | R | - |
|  | To Determine the Safety Tolerability Pharmacokinetics and Effect on Pain of a Single IA Administration of Canakinumab in Patients With KOA | II | NCT01160822 | C |  |
| LNA043 | A Safety and Efficacy Study of Anti-inflammatory (Canakinumab) and Cartilage Stimulating (LNA043) Drugs Injected Into the Knee Joint of Participants With KOA (OA) | II | NCT04814368 | R | [134] [135] |
|  | Study of Efficacy Safety and Tolerability of LNA043 in Patients With KOA (ONWARDS) | II | NCT04864392 | R |  |
|  | Study of Safety Tolerability Preliminary Efficacy of IA LNA043 Injections in Patients With Articular Cartilage Lesions and KOA. | II | NCT03275064 | A |  |
|  | Study of Safety Tolerability and Pharmacokinetics of LNA043 in Japanese OA Participants | I | NCT04564053 | C |  |
|  | First-in-human Single Ascending Dose Study of LNA043 in Patients Scheduled for Total Knee Replacement | I | NCT02491281 | C |  |
| TABLE 1 \| (Continued) Clinical Trial of IA-based therapeutic for the treatment of osteoarthritis. | | | | | |
| Biological / chemical compound for therapy | **Condition** | **Phase** | **ClinicalTrials.gov Identifier** | **Recruitment Status** | **Ref.** |
| PSC-01 | Evaluation of Safety and Exploratory Efficacy of an Autologous Adipose-derived Cell Therapy Product for Treatment of Single KOA | I | NCT04043819 | A | - |
| CNTX-4975-05 | An Open-label 8-week Safety Efficacy and Assessment of Cooling Methods for Administration of CNTX-4975-05 for Knee OA | III | NCT03661996 | C |  |
|  | A Study to Compare Levels of Capsaicin After IA Injection and Topical Application in Patients With Painful KOA | I | NCT03576508 | C |  |
|  | A Clinical Study to Test Efficacy and Safety of Repeat Doses of CNTX-4975-05 in Patients with OA Knee Pain | III | NCT03660943 | C |  |
|  | A Phase 3 Efficacy and Safety Study of IA CNTX-4975-05 (Capsaicin) vs Placebo in Subjects with OA Knee Pain | III | NCT03429049 | C |  |
|  | The Effect of Injection Site Cooling on Pain Experienced After the Administration of CNTX-4975-05 Into the Knee | I | NCT03472677 | C |  |
| StroMel | Safety and Tolerability of StroMel™ in Subjects with Moderate to Severe KOA Joint | I/II | NCT04750252 | Not yet R | - |
| V120083 | A Study to Assess the Analgesic Efficacy and Safety of V120083 in Subjects with OA (OA) of the Knee | II | NCT03028870 | C | - |
| FX201 | The evaluation of the Safety and Tolerability of FX201 in Patients with KOA | I | NCT04119687 | A | - |
| CNTX-4975-05 | An Open-label 8-week Safety Efficacy and Assessment of Cooling Methods for Administration of CNTX-4975-05 for Knee OA | III | NCT03661996 | C |  |
|  | A Study to Compare Levels of Capsaicin After IA Injection and Topical Application in Patients with Painful KOA | I | NCT03576508 | C |  |
|  | A Clinical Study to Test Efficacy and Safety of Repeat Doses of CNTX-4975-05 in Patients with OA Knee Pain | III | NCT03660943 | C |  |
|  | A Phase 3 Efficacy and Safety Study of IA CNTX-4975-05 (Capsaicin) vs Placebo in Subjects with OA Knee Pain | III | NCT03429049 | C |  |
|  | The Effect of Injection Site Cooling on Pain Experienced After the Administration of CNTX-4975-05 Into the Knee | I | NCT03472677 | C |  |
| TLC599 | Single-Dose Administration Trial of TLC599 in KOA | I/II | NCT02803307 | C | [136] |
|  | A Phase 2 Open Label PK Study of TLC599 in Subject With KOA | II | NCT03754049 | C |  |
|  | Extended and Controlled Release Liposomal Formulated Dexamethasone for Chronic Knee OA Pain (EXCELLENCE) | III | NCT04123561 | C |  |
|  | Phase IIa Randomized Double Blinded Placebo Controlled Dose Finding Study for TLC599 in OA Patients | II | NCT03005873 | C |  |
| TABLE 1 \| (Continued) Clinical Trial of IA-based therapeutic for the treatment of osteoarthritis. | | | | | |
| Biological / chemical compound for therapy | **Condition** | **Phase** | **ClinicalTrials.gov Identifier** | **Recruitment Status** | **Ref.** |
| X0002 | Assess Efficacy and Safety of X0002 in Treatment of KOA | III | NCT05324163 | R | - |
|  | To Assess Efficacy and Safety of X0002 in Treatment of KOA | II | NCT03691818 | C |  |
|  | A The evaluation of the Efficacy and Safety of X0002 Spray in Subjects with OA Knee Pain | III | NCT03081806 | R |  |
|  | A The evaluation of the Efficacy and Safety of X0002 Spray in Subjects With OA | II | NCT02067611 | C |  |
| MEDI7352 | A Study of the Efficacy and Safety of MEDI7352 in Subjects with Painful KOA (BESPOKE) | II | NCT04675034 | R | - |
|  | To Assess Safety Tolerability Pharmacokinetics and Immunogenicity of MEDI7352 in Healthy Volunteers | I | NCT04770428 | C |  |
|  | A Study of MEDI7352 in Painful KOA | I | NCT02508155 | C |  |
| Resiniferatoxin | The evaluation of IA Resiniferatoxin to Treat Moderate to Severe Pain From KOA | II | NCT04885972 | R | [137] |
|  | The evaluation of Resiniferatoxin in Patients With KOA Whose Total Knee Replacement Surgery is Delayed | III | NCT04386980 | W |  |
|  | A Phase 3 The evaluation of the Efficacy and Safety of Resiniferatoxin for Pain Due to KOA | III | NCT04044742 | W |  |
|  | Study of Resiniferatoxin for Knee Pain in Moderate to Severe OA | I | NCT03542838 | C |  |
| AMZ001 | AMZ001 for the Treatment of KOA Symptoms | II/III | NCT03691844 | C | [138] [139] |
| 2ccPA | 2ccPA Study in Patients With Symptomatic KOA | I | NCT04229394 | C | - |
| XT-150 | Efficacy and Safety of XT-150 in KOA | II | NCT04124042 | C | [140] |
|  | Follow on Extension of XT-150-1-0201 | I | NCT03769662 | C |  |
|  | Safety Tolerability and Efficacy of XT-150 for the Treatment of Osteoarthritic Pain | I | NCT03477487 | C |  |
|  | Preliminary Evaluation of Safety Tolerability and Efficacy of XT-150 for the Treatment of Osteoarthritic Pain | I | NCT03282149 | C |  |
| YYD302 | Clinical Trial of YYD302 (Phase3) for Treatment of KOA | III | NCT03561779 | C | [141] [142] |
|  | Clinical Trial of YYD302 for Treatment of KOA | I/II | NCT02965495 | C |  |
| LRX712 | First-in-human Safety Tolerability and Pharmacokinetics Study of LRX712 in Osteoarthritic Patients | I | NCT03355196 | C | - |
|  | Safety Tolerability and Preliminary Efficacy of Multiple IA Injections of LRX712 in Patients With Knee OA | II | NCT04097379 | R |  |
| TABLE 1 \| (Continued) Clinical Trial of IA-based therapeutic for the treatment of osteoarthritis. | | | | | |
| Biological / chemical compound for therapy | **Condition** | **Phase** | **ClinicalTrials.gov Identifier** | **Recruitment Status** | **Ref.** |
| Celltex- AdMSCs | Autologous Adipose Tissue-Derived Mesenchymal Stem Cells (AdMSCs) for OA (AdMSCs) | II | NCT04448106 | Not yet R | - |
| Lopain | IA Lopain (MTX-071) Phase I/IIa Study in Chronic Osteoarthritic Knee Joint Pain | I | NCT02566564 | C | - |
| MT-5547 | Efficacy and Safety of MT-5547 in Patients with OA Accompanied by Moderate to Severe Pain | II/III | NCT03245008 | C | - |
| FX006  (Zilretta) | The assessment of the Safety of Repeat Administration of FX006 Administered to Patients With KOA | III | NCT03046446 | C | [143] [144] |
|  | Study of FX006 for the Treatment of Pain in Patients With KOA | III | NCT02357459 | C |  |
|  | Study of FX006 vs Normal Saline in Patients With KOA | II | NCT02116972 | C |  |
|  | Study of FX006 in Patients With KOA | II | NCT01487161 | C |  |
|  | Pharmacokinetic and Pharmacodynamic Study of FX006 in Patients With KOA | II | NCT01487200 | C |  |
|  | Proof of Concept Study Comparing FX006 to Kenalog®-40 in Patients With Post-Traumatic KOA | II | NCT02468583 | T |  |
|  | Study to Characterize the Local Duration of Exposure From FX006 in Patients With KOA | II | NCT02003365 | C |  |
|  | Study to Characterize the PK and Local Extent and Duration of Exposure From FX006 in Patients With KOA | II | NCT02637323 | C |  |
|  | Study to Compare Exposure of TA Following Administration of Either FX006 or TAcs in Patients With Bilateral Knee OA | II | NCT03378076 | C |  |
|  | The evaluation of the Effect of FX006 on Synovial Inflammation in Patients With KOA | III | NCT03529942 | C |  |
